# Dr. Edwards’ Report—Patent Medicines

**Published:** 1849-07

**Authors:** 


### Part 4.—Editorial.

### ARTICLE I.

#### REPORT OF DR. EDWARDS---PATENT MEDICINES.

We have had occasion heretofore to bear testimony to the valuable service rendered to the profession by this gentlemen, from his place in Congress. As a general thing we dislike to see a physician deserting the profession for the purpose of embarking in politics, but in this case we are obliged to suppose that Dr. Edwards has accomplished more for the profession and the community than he could have done merely as a general practitioner. So far as we now recollect, he is the only member of Congress who has ever undertaken to vindicate the claims of physicians to the respect of the government, or to protect the nation from the gross imposition of the nostrum venders. He deserves the highest praise for his bill to prevent the introduction of spurious drugs and no less for his later effort to strip off the cloak of mystery under which the quack has so long practised upon the credulity of the multitude.

After a careful perusal of this report, and some reflection upon it, we give it as our decided opinion that of all the unprincipled knaves who prey upon mankind, the nostrum monger is chief. The sharp-sighted rascal who palms off his wares at a false estimate is simply contemptible: the quack who trifles with human health is most detestable. Nostrum mongers may be fitly divided into three classes---1st, those who put out innocuous and inert compounds, such as Bristol's Sarsaparilla, Dow's family medicine, etc.; 2d, Those whose nostrums possess some medicinal powers in which the inventor himself, generally an ignorant person, has confidence, deceiving himself as well as the community; 3d, and most wicked of all, the unprincipled person who obtains a powerful prescription, which should never be administered except under the superintendence of a competent physician, carries it to the Patent Office, swears it is new and valuable and under the broad seal of the national government, scatters it abroad to the serious detriment of thousands. For the accomplishment of their design, this class does not stick at the most infamous perjury. As an instance of this, look at the *vegetable* cancer powder, whose composition is detailed on page 24 of the report. This nostrum is composed of seven parts caustic potash and one part sesquioxide of iron. This imposition was rather glaring, and a patent was refused.

A modest man is struck aghast at the impertinence and assurance of these gentry, to say nothing of their respect for the moral law. Take the following as a sample---the latin contained in which is, as Dominie Sampson would say, "prodigious:" "Thurston's Anti-Fever Pills: Prescription thereof viz.: sulph quinine, three ounces; rad. rhei pulveris, three ounces; *piperinal plural*, one ounce; acidum *sulphuriam* dilutum, (quantam sufficit) *formam massam*. Make the above recipe into pills of three and a half grains each." The italics are our own, and our conscience almost chides us that we have not directed capitals instead, for we presume our readers will agree with us that the recipe evinces a genius capable of producing much more remarkable results in philology than pharmacy. But this great skill in the classics availed nothing. The commissioner chanced to know that quinine had been before used in the treatment of intermittents, and refused a patent. Here is another *new* and useful invention. John E. McClure's preparation for the cure of gonorrhœa: "Oil of cubebs, three drachms; spts. nitre dulc., six drachms; bals. copaiba, one and a half ounces: simple syrup, two ounces; comp. spts. *lavendula*, three drachms; tinc. cantharides, one and a half ounces; oil balsam copaiba, one and a half drachms; *theberiac* tincture [this we suppose is an effort at thebaic tincture, the old term for tinct. opii,] one and a half drachms." Unfortunately for the purse of this personage, the United States declined giving him the exclusive right to vend an old and well known prescription, and extort money from the *unfortunate females* and young bloods of the large towns and cities. Another asks a patent for enclosing anthelmintic medicines in gelatine capsules.

Conspicuous on this calendar of quacks, stand the names of Samuel Thompson and Horton Howard, the apostles of the steam system as it has been called, and which, like other steam machinery, is giving indications of blowing up its inventors. This miserable cheat also came forward and requested the government to throw over it the mantle of protection, and give it a monopoly of gum myrrh, capsicum, and lobelia, and, strange to relate, the Patent Office acceded to the request, giving the impudent mountebank an indorsement which enabled him to compete with competent physicians.

The stronghold of the nostrum is the mystery in which it is enveloped. Mankind are prone to reverence what they do not understand, and the nostrum monger thoroughly appreciates that familiarity begets contempt. So soon as the composition of the nostrum is known, the charm is broken, and the public are ready to swallow down something else, it makes little difference what, provided they do not know what it is. The man who is cured of the ague by quinine disguised as Sappington's Pills, is enraged at the cheat which has been practised upon him, but is ready to swallow the same drug disguised under another name, such, for instance, as Smith's Tonic Syrup. So soon as any quack remedy obtains reputation, apothecaries and pharmaceutists analyze and imitate it, the secret of its preparation leaks out, and its popularity is at an end. This was the case with Morrison's Pills, Godfrey's Cordial, Bateman's Drops, and divers other villainous compounds, which are now forgotten, or remembered only as serving to illustrate the gullibility of mankind. In all efforts to free the community from these nuisances, attention must be directed to dispelling the mystery which lends them their magical influence. It would be needless to undertake to suppress them by legislativ enactment. This country is entirely too free for that. It may do for the subjects of kings to have their lives protected by law from the attacks of charlatans, but in this free country we insist not only on the right of living as we please, but of dying as we please.

The quack has many allies in his business. There are many editors who are ignorant of the plainest principles of the science of medicine, anatomy, or physiology, and others who think they know something and desire to evince their knowledge by a fling at the profession. These papers have a greater or less influence over their readers. The quack gives the editor a box of his excellent purgative pills or a bottle of his invaluable vermifuge, pays him liberally for advertising, and gets in return a laudatory paragraph in the editorial column. Of course this is not general amongst the gentlemen of the press, but we could name many widely circulated and influential journals which puff quack medicines, and are open advocates of hydropathy, homoœpathy, Thompsonianism, etc. Ministers of the gospel, too, are often found ranged on the side of the quack; lending their powerful aid to the support of innovation upon the medical profession, though there is no body of men so jealous of encroachment upon their own. The certificate of a respectable minister is of great value to the nostrum mongers, and is cheaply obtained at the price of a bottle of tar water, or solution of tartarized antimony, scented with aromatics. The quack shrewdly concludes that if he can secure the shepherd, the sheep will not be slow to follow. Druggists and apothecaries all over the country are extensively engaged in the business of vending patent medicines, We suppose there is hardly an establishment of this kind in the country that is not largely embarked in this despicable business. One cannot pass along the streets of our larger towns or cities without being stared in the face by flaming hand bills, descriptive of the virtues of some wonderful electuary or magical pain killer. Of course this business yields large profits on the capital employed. The proprietor can afford to give the druggist a commission of from twenty-five to fifty per cent., and still have a handsome profit for himself. We do not accuse apothecaries of being actuated in this matter solely by cupidity, for we cannot believe they would, as a body, trifle with the health of their customers merely for money. The error arises partly from ignorance and partly from thoughtlessness, though it is not to be disguised that there are some who embark in the business solely for the profit. We have known the following trick performed again and again: An apothecary has labels struck for Brandreth's Pills, Peter's Pills, or any other that chance to be the rage at the time; he then makes a batch of pills, say the compound cathartic pills of the dispensatory, and fills all the boxes from the same mass. Of course he is much entertained to hear a discussion amongst his customers as to the relative merits of Brandreth and Peters or the superiority of scammony and colocynth over colocynth and scammony. Another stronghold of the patent medicine is the countenance and support of the government. We believe it is right and just that government should protect the mechanic arts, and that the profit of new and useful inventions should accrue to the inventor. But physicians ask no exclusive property in any thing that pertains to the healing art, and promptly denounce as an empiric or pretender any one who makes a mystery of anything that might have an application to the treatment of disease, or the alleviation of suffering. Mystery in medicine is *prima facie* evidence of quackery, and it is unaccountable to us that the patent office has never learned this fact. So long as the national authorities place the quack medicine in the same rank with the cotton gin or the improvements in the steam engine, as a new and useful invention, it is not to be wondered at that newspapers will puff, that parsons will give certificates, that apothecaries will vend or that the ignorant and hypochondriac will swallow them. Commissioners of patents, we presume are qualified to judge of improvements in the arts, but it is not at all derogatory to them to suppose that they are not competent judges of what is new and useful in medicine; and we suggest that it would be proper for them either to refuse patents for alleged improvements in medicine; or, what would amount to the same thing, refer all such applications to a board of intelligent physicians. We understand that Dr. Edwards introduced a bill prohibiting the issue of such patents, which passed the house of representatives, but failed in the senate for want of time. We sincerely hope the bill will be taken up and acted upon at an early day of the ensuing session. Let the national government at least refuse to lend its influence and countenance to these vile impositions. Here is the first step to be taken by the profession in the suppression of quack nostrums, viz., to unite in the effort to make the provisions of Dr. Edwards' bill a law.

We have in a previous number of this Journal had occasion to notice a bill brought before the legislature of Wisconsin. This bill required every physician to send a written prescription to accompany the medicine administered to his patients. To this we have no objection, for the scientific physician is not afraid of scrutiny; but we would have the same law apply to the quack medicine. We would have an exact detail of its composition accompany every box and bottle, and surely the nostrum vender cannot complain at being put upon the same level with the regular practitioner. With regard to that portion of the press that lends its influence to these cheats, the profession has little to hope: they are not to be reasoned out of their conceit that they are immensely wise, and perfectly well qualified to teach a difficult and abstruse science of which they have the merest smattering; or they are so wretchedly bigoted as to be beyond the reach of appeal or argument. To the praise of the clerical profession be it said, we know many who resolutely oppose the wholesale knavery practised upon the community by the patent medicine men; and we believe that those who are entrapped into giving certificates, err more from thoughtlessness than from reprehensible motives. To such it is only necessary that physicians should point out the error which they have committed, and they will abandon it. The most difficult ally of the nostrum monger with whom the physician has to contend, is the druggist and apothecary. Many persons are in the habit of calling upon them for prescriptions, and get some alleged panacea, which purports to cure the disease with which they are afflicted. There are two methods of remedying the evil at this point. In the first place apothecaries, particularly in cities, should be made to understand that they constitute a branch of the profession, and as such should feel themselves above the contemptible occupation of vending quack specifics, or lending their influence to assist the nostrum monger in his despicable business of cheating the simple; or if they cannot be made to understand this, or prefer profit to honor, it is the duty of the profession of their neighborhood to denounce them promptly, and forego all dealings with them. As things now stand apothecaries can hardly be held reprehensible; for the profession has taken no combined action on the subject; but when once the profession takes the matter seriously in hand, we are confident they can induce the majority of the apothecaries to coincide with them in sentiment, and can bring such influence to bear on the refractory as to render them willing to come to terms. On a full view of the subject this evil does not strike us as an irremediable one. If the profession can detach the national goverment from the nostrum-vender by the passage of Dr. Edwards bill, and can procure the passage through the state legislatures, of laws compelling these gentry to attach to each bottle and box, an exact detail of the compenent parts of the nostrum, we think the nuisance will be pretty well abated, or so far at least as to place it within the power of the physician to give it the *coup de grace.*

The public are most grossly imposed upon by certificates of wonderful cures accompanying these nostrums, any one of which is equally celebrated for curing the most opposite diseases known to our nosology. It may not be inappropriate to drop a hint on this subject, which may be of use to the very youngest members of the medical profession, and put them on their guard against being imposed upon in this way. The quack seeks out some hypochondriac, assures him he has a specific for his complaint; the simpleton places great confidence in the impostor, swallows the potion, feels immensely relieved, and is willing to give the most extravagant certificate of the efficacy of the medicine. Again, many persons feel slightly indisposed, not sick enough to go to the doctor; and fancy that "old Dr. Townsends Sarsaparilla" is just the thing they need, and straightway hasten to the drug store and get a huge bottle of this villanious compound of Sarsaparilla, Pipsissewa, Dock roots, and enough of the "critter" to give it a flavor. They are cured of course and feeling grateful to heaven and "old Dr. Townsend," are ready to give the most extravagant certificate, which is inserted, in prominent type, in the next day's paper, stating that the highly respectable Mr. McSpadgen, was cured of dyspepsia, scrofula, liver complaint, and consumption, by one bottle of this invaluable remedy. These certificates always remind us of the Irishman who went to a physician to consult about his wife. The doctor gave him a blister, telling him it was to be put on her chest. Meeting Paddy a few days afterward, the doctor inquired after the woman's health, and learned that she had entirely recovered. "You put the blister on her chest, did you Patrick?"

"No doctor, we have no chest, but I put it on the lid of an old trunk, and found it answered just as well, for she began to get better from that hour. Oh its a mighty great medicine is the blisther entirely."

Think you Paddy would not have given a tremendous certificate of the efficacy of the blister on the *chest* or on the *trunk?*

The public does not, as a general rule, rightly appreciate the motive which induces the physician to denounce nostrums. It is thought by many that the quack medicine diminishes the fees of the profession. This is, we suppose, a mistake, if the nostrum is detrimental to the health of the community, of which there is no doubt, it is hardly to be supposed that the physician's fees will not be augmented. We will give an instance in point.

A man contracts syphilis; to avoid expense he buys some reputed specific for which he pays one or two dollars; while using this the disease extends its ravages, the system becomes contaminated, and the patient is compelled to place himself under the direction of a competent medical man, and undergo a course of treatment involving a great sacrifice of time, and accompanied by a heavy bill of fees. Had he applied in time, the application of a stick of caustic would have removed the disease at a trifling expense, and saved him a vast amount of inconvenience and suffering. This is not an isolated, case and will serve to illustrate the position assumed above. The objection of the profession rests on other grounds. The nostrum is a satire upon the profession of medicine, and so far as successful has a tendency to bring our noble science into contempt with the intelligent, discerning portion of the community. Of course this is an aggression upon our honor not to be tamely borne. The dignity of our profession demands that it should be resented. Physicians are the regularly constituted overseers of all that pertains to the health of the community in which they reside, and it is their imperative duty to set their faces resolutely against anything that is judged to have a pernicious tendency upon the physical well being of their patrons. These are the grounds of objection to the quack medicine. If any nostrum is known to be really vauable, the regular profession never hesitate to analyse and imitate it in their prescriptions, for there is no patent law that would prevent them from so doing.

We commend Dr. Edwards' report to our readers, hoping that each one will peruse it for himself, and circulate it amongst his friends, as we are confident that the diffusion of knowledge on this subject, will go far to rid the community of the rascally impositions practiced by the nostrum monger.

